# Notes and News

**Published:** 1903-08-22

**Authors:** 


### Notes and News.

It is proposed to erect a general hospital for the United States Army at Washington.

Last week fifteen cases of plague were reported from Mauritius of which twelve terminated fatally.

The late Mr. Matthew Whiting, of Clapham, has bequeathed £5,000 to the Bolingbroke Hospital, Wandsworth, and large sums to other hospitals.

A fine of £5 and £10 10s. costs has been imposed upon Parke's Drug Stores, Camden Town, for the sale of so-called "Blaud's Pills," which were deficient in ferrous carbonate to the extent of 78·5 per cent.

By the death of Dr. William Playfair, at a period of life when many of his contemporaries are still placing the fruits of their ripe experience at the service of the public, and when others are reasonably expecting a period of ease and tranquillity at the close of their professional labours, the medical profession has lost one of its most striking figureheads. Born in 1836, he received his education at St. Andrews and Edinburgh. Shortly after entering the medical profession he proceeded to India, where he served as assistant surgeon in the Bengal Army during 1857, and afterwards, for two years, held the post of Professor of Surgery in the Medical College, Calcutta. He subsequently returned to England and commenced to practise as a physician in London. At the time of his death he was Emeritus Professor of Obstetric Medicine and Consulting Physician for Diseases of Women and Children at King's College Hospital, Consulting Physician to the General Lying-in Hospital, and to the Evelina Hospital for Sick Children. Latterly he had retired from active practice, and resided in the country. The funeral took place privately on Saturday, August 15th, at St. Andrews, where Lord Playfair and Sir Lambert Playfair are also buried.

There are now 62 small-pox patients under treatment at Cambridge.

The late Mr. William Cadge, Consulting Surgeon to the Norfolk and Norwich Hospital, has left £5,000 to that institution and £1,000 to the Royal Medical Benevolent College.

In a letter to the *Times*, Major Ross draws attention to a report from Major Penton, principal Medical Officer of the Sudan, showing the importance of a steady crusade against the mosquito. In respect to Ismailia, since measures to exterminate have been adopted, malarial fever has dropped to three cases in hospital from 52 cases during the previous year, and the number of fever cases fell from 569 to 72 in the same period.

The possibilities of infection which rats are responsible for have been clearly illustrated in Sydney. The City Councils' Health Committee have kept a close watch on the haunts of rats since the first appearance of plague in the City. Large numbers of rats from all parts of the City are examined, and recently two were found infected with plague. This it is believed had been contracted from an outside source, and the evil discovered before the infection was communicated to human beings.

It is a reflection upon the management of the St. Pancras Infirmary that it was left to a jury to make the following recommendation, that "No patient kept in the mental wards for the purpose of observation should be allowed to be employed at any work outside the mental ward without the authority of the medical officer." The necessity for this elementary precaution is so obvious that it is difficult to understand how it could have been disregarded. At St. Pancras failure to observe this precaution resulted recently in the death of a man who committed suicide when assisting in the workhouse kitchen.

We learn that the Liverpool School of Tropical Medicine has decided to despatch a trypanosoma expedition to the Congo Free State next month. The expedition has been arranged with the co operation of the Government of the Congo Free State. The objects of the expedition will be to report on the sanitary conditions of Boma, Leopoldville, and other important centres visited, and to make recommendations for the improvement of existing sanitary conditions. Secondly, to continue the work of trypanosomiasis, human and animal, begun in the Gambia by Dr. Dutton in 1901, and continued for the past 12 months in the Gambia and Senegal by Dr. Dutton and Dr. Todd, including the occurrence and distribution of trypanosoma in the Congo, the carriers of the parasite, and the relation of trypanosoma to sleeping sickness. The expedition is under the charge of Dr. J. E. Dutton, and consists of Dr. Dutton, Dr. J. L. Todd, and Dr. C. Christy. It will start for the Congo by the steamship *Albertville*, every facility for travel having been placed at their disposal by Sir Alfred Jones, chairman of the school. The Congo Free State have contributed generously towards the expenses of the expedition, which will be heavy.

